# Aphantasia and psychological disorder: Current connections, defining the imagery deficit and future directions

**DOI:** 10.3389/fpsyg.2022.822989

**Published:** 2022-10-14

**Authors:** Dan Cavedon-Taylor

**Affiliations:** The Open University (United Kingdom), Milton Keynes, United Kingdom

**Keywords:** addiction, aphantasia, intrusive imagery, maladaptive daydreaming, mental imagery, post-traumatic stress disorder, SUIS, VVIQ

## Abstract

Aphantasia is a condition characterized by a deficit of mental imagery. Since several psychopathologies are partially maintained by mental imagery, it may be illuminating to consider the condition against the background of psychological disorder. After outlining current findings and hypotheses regarding aphantasia and psychopathology, this paper suggests that some support for defining aphantasia as a lack of voluntary imagery may be found here. The paper then outlines potentially fruitful directions for future research into aphantasia in general and its relation to psychopathology in particular, including rethinking use of the SUIS to measure involuntary imagery, whether aphantasia offers protection against addiction, and whether hyperphantasia is a potential risk factor for maladaptive daydreaming, among others.

## Introduction

A growing body of literature suggests that dysfunctions of mental imagery make significant contributions to a variety of psychopathologies (Pearson et al., 2013, 2015; Çili and Stopa, 2015). A small sample includes the following:

- *Post-traumatic stress disorder (PTSD)*. PTSD may involve ‘re-experiencing’ past trauma *via* involuntary, imagery-based flashbacks. Indeed, the occurrence of such images is among the condition’s diagnostic criteria in the DSM-5 as well as a postulated cause of PTSD’s characteristic hyperarousal (Solomon et al., 2009; McTeague et al., 2010).
- *Major depressive disorder*. A number of studies have evidenced the existence of intrusive, imagery-based memories in depressed individuals, with up to 87–96% of patients reporting flashbacks to negative life-events (Brewin et al., 1996; Newby and Moulds, 2011). Moreover, when at their most hopeless many depressed individuals report future-oriented, imagery-based ‘flashforwards’ to acts of suicide and self-harm (Crane et al., 2012). Insofar as negative imagery can be expected to contribute to low mood, a vicious cycle may occur in which negative imagery causes low mood that triggers more negative imagery, prompting mood to lower further still, and so on (Weßlau and Steil, 2014).
- *Social phobia*. Socially phobic individuals frequently report intrusive, negative images in social situations. Sometimes these are imagery-based memories of negative social interactions from the patient’s past (Hackmann et al., 2000). But such imagery intrusions may also represent the patient as they are believed to appear now, ‘looking foolish,’ in the eyes of their present interlocutor (Clark and Wells, 1995; Hirsch et al., 2003).
- *Bipolar disorder*. A leading model in the clinical literature, ‘the mood amplification model,’ identifies vivid, intrusive, future-oriented imagery as the mechanism whereby an initially positive moods spirals into mania or hypomania (Holmes et al., 2008; Ivins et al., 2014; Ng et al., 2016; O’Donnell et al., 2018). These future-oriented images may be considered akin to the flashforwards suffered by those with severe depression (Crane et al., 2012), but involve imagery of positive and exciting future possibilities rather than negative and traumatic ones.

Imagery dysfunctions, especially related to excess vivacity or intrusivity, have also been hypothesized to shape the episodes of conditions as diverse as schizophrenia (Aleman et al., 2005; Kang et al., 2011), obsessive–compulsive disorder (de Silva, 1986; Speckens et al., 2007) and anorexia nervosa (Gadsby, 2017; Selby and Coniglio, 2020).

It would be a mistake to think that any of these disorders can be explained monolithically, in imagery-based terms alone. Moreover, theories linking mental imagery to psychopathology are at odds with models of both psychological disorder and mental architecture that make no reference to mental imagery (O’Regan and Noë, 2001; Pylyshyn, 2002). However, insofar as intervening on the relevant imagery dysfunctions is increasingly recognized as an effective treatment of these disorders (Smucker et al., 1995; Brewin et al., 2009; Arntz, 2012; Holmes et al., 2016), this is a significant reason to affirm both the reality of mental imagery and, crucially, that imagery’s relation to psychopathology goes beyond that of mere association or epiphenomenal effect. In terms of the nature of such interventions, these include reducing the vivacity of a patient’s intrusive imagery, replacing negative imagery with more positive images, altering the patient’s reaction to their imagery, or reducing the frequency of intrusive imagery (see Hackmann et al., 2011 for an overview).

These findings are likely to be informative for our understanding of aphantasia (Zeman et al., 2015; Zeman, 2020). Aphantasia is a deficit of mental imagery that can occur both congenitally and non-congenitally. Although reports of the condition date to at least the late 1800s (Galton, 1880; Charcot, 1889), sustained attention to aphantasia is relatively new, with recent research attempting to shed light on its relation to autism (Dance et al., 2021), career choice (Zeman et al., 2020), dreaming (Dawes et al., 2020), emotions (Wicken et al., 2021), memory (Jacobs et al., 2018), perceptual processing (Keogh and Pearson, 2018), shared vision/imagery neural substrates (Dijkstra et al., 2019; Bainbridge et al., 2021; Cavedon-Taylor, 2021) and synesthesia (Dance et al., 2021).

The primary measure used to identify aphantasic individuals is self-reports, i.e., low scores on the Vividness of Visual Imagery Questionnaire (VVIQ; Zeman et al., 2010). The VVIQ asks patients to conjure various mental images (‘visualize a rising sun’) and rate their clarity and vividness relative to normal vision. More recently, at least two psychophysical correlates of aphantasia have been identified and which may be used to confirm its presence more objectively. These include a reduced imagery-based priming effect on vision in conditions of binocular rivalry (Keogh and Pearson, 2018) and reduced autonomic system arousal when reading emotionally-charged texts (Wicken et al., 2021).

While it is agreed that the imagery capacities of aphantasics are severely attenuated, the existing literature equivocates on the exact nature of that attenuation. Aphantasics have been variously claimed to lack metacognitive access to mental imagery (Nanay, 2021), lack voluntarily-generated mental imagery (Zeman et al., 2015; Pounder et al., 2022) and lack mental imagery altogether (Keogh and Pearson, 2018; Wicken et al., 2021). These are all quite different claims, however, and they have differing implications for our understanding of the condition. The first pictures aphantasia as a deficit of introspection, the second as a deficit of volition and the third as a thoroughgoing lack of imagery capacity. The question of which definition is preferable is addressed only rarely (Keogh and Pearson, 2018; Nanay, 2021).

After outlining current findings and hypotheses regarding aphantasia and psychopathology (“Aphantasia and psychological disorder: Current connections”), this paper goes on to enquire into what each account of the condition predicts in terms of the susceptibility of aphantasics to psychopathology, suggesting that some support for the volitional definition of aphantasia, i.e., as a lack of voluntary imagery, may be found here (“Defining the imagery deficit”). The paper then outlines potentially fruitful directions for future research into aphantasia and psychopathology (“Future directions”), including a link between aphantasia and addiction and between hyperphantasia and maladaptive daydreaming.

## Aphantasia and psychological disorder: Current connections

If imagery dysfunctions contribute to a wide range of psychological disorders and aphantasia is a deficit of imagery, then it might be suggested that aphantasia constitutes a benefit in terms of protecting against psychopathology. This was among the hypotheses of one of the first, large-sample studies of aphantasic individuals (Dawes et al., 2020). Puzzlingly, the study failed to confirm that hypothesis. In terms of mental illness history, trauma-based psychopathology in particular, no significant difference was found between an aphantasic sample and a non-aphantasic control group. This result was yielded using the PCL-5 (Weathers et al., 2013), a 20-item self-report measure used in the DSM-5 concerning outlook on past adverse life-events and the current prevalence of intrusive memories. Although Dawes et al. (2020) caution against concluding from this result that aphantasics receive no benefit from their condition in terms of a reduced susceptibility to psychopathology, the comorbidity of aphantasia and psychological disorder may not be an anomaly. Indeed, some results, although mostly anecdotal, are suggestive of a causal influence between the two.

For one, a causal influence from aphantasia to psychological disorder may be possible: for one patient, being congenitally aphantasic seemingly led to poor work performance and eventual reactive depression (Takahashi and Gyoba, 2021). The reverse influence, from psychological disorder to aphantasia, may also be possible. Indeed, some have argued in support of this connection on the basis of historical analyses of early reports of imagery deficits in patients with derealization, depersonalization and depression (Zago et al., 2011; de Vito and Bartolomeo, 2016). More recent studies of patients with depersonalization disorder similarly point to a potential contribution that psychological disorders can make to a deficit of imagery (Lambert et al., 2001; Phillips et al., 2001). In light of these historical analyses, Adam Zeman (Zeman, 2020) who first coined the term ‘aphantasia,’ has supported distinguishing within the category of non-congenital aphantasia ‘neurological aphantasia’ (aphantasia caused by brain damage) from ‘psychogenic aphantasia’ (aphantasia caused by psychological disorder). From a clinical perspective, it may not always be a simple matter to tell congenital and non-congenital aphantasia apart. For instance, someone with non-congenital aphantasia of a psychogenic origin may have acquired the condition as a result of trauma, with their lack of imagery being a coping mechanism, one that also causes them to forget that they ever had imagery in the first place. Such a person would have non-congenital aphantasia yet mistakenly think that they have been aphantasic from birth.^1^

As mentioned above, at least three different accounts of aphantasia can be found in the literature: aphantasia as a lack of metacognition, aphantasia as a lack of voluntary mental imagery and aphantasia as a thoroughgoing lack of imagery capacity. Crucially, each makes varying predictions about the susceptibility of aphantasics to psychopathology. These predictions can be assessed in light of the findings of this section. Since research into aphantasia and psychopathology, like research into aphantasia in general, remains in its infancy, some caution is necessary here. At this early stage in our understanding of the condition, this paper’s conclusions are necessarily tentative.

## Defining the imagery deficit

Although aphantasia is considered a deficit of imagery, a rival hypothesis is that it is a metacognitive deficit. This account is sometimes stated in the literature only to be swiftly rejected (Keogh and Pearson, 2018), but it has recently received some tentative support (Nanay, 2021). The account is ambiguous on the nature of the deficit. While it is most naturally read as saying that aphantasia is really a deficit of introspection or access, it might also be understood as claiming that aphantasics lack a capacity for conscious imagery. If there is such a thing as unconscious imagery (Brogaard and Gatzia, 2017), both disambiguations share a crucial commitment to the claim that aphantasia leaves this form of imagery intact.

The metacognitive account predicts that aphantasics are significantly less likely to suffer from the psychopathologies canvassed in “Introduction” insofar as the imagery-based dysfunctions discussed there seemingly contribute to those psychopathologies by virtue of being conscious. For instance, the traumatic imagery undergone by patients with, e.g., PTSD, depression and social phobia, are only likely to cause distress to the patient if actually experienced. Similarly, when it comes to the future-oriented imagery of exciting possibilities undergone by patients with bipolar disorder, these are only likely to amplify an initially positive mood if consciously accessed. As mentioned, Dawes et al. (2020) caution against the view that aphantasics receive no benefit from their condition in terms of protection against psychopathology. But the fact that their aphantasic group and non-aphantasic controls exhibited scant difference in terms of incidence of trauma-based psychopathology, and that causal influence between aphantasia and psychopathology may run in both directions, is preliminary evidence against the metacognitive view insofar as it entails that aphantasia constitutes a barrier to psychopathology.

A very different account of aphantasia understands it as a lack of voluntarily-generated mental imagery (Zeman et al., 2015; Pounder et al., 2022); recall from “Introduction” that the VVIQ initially used to confirm an imagery deficit in aphantasics relies on the subject conjuring imagery voluntarily. On this account, aphantasics have mental imagery, both conscious and unconscious, they just lack one way of generating it. This view pictures aphantasia as primarily an absence of volition with respect to imagery and only partially an absence of imagery itself.

Crucially, and unlike the metacognitive account, the volitional account seems to predict that aphantasics are likely to be as susceptible as non-aphantasics to conditions like PTSD, depression, social phobia and bipolar disorder. This is because the imagery-based flashbacks, and flashforwards, reportedly contributing to the maintenance of these conditions occur involuntarily, ‘intrusively.’ And it is this kind of imagery that the volitional account says remains intact in aphantasia. Indeed, in one study on congenital aphantasia, the majority of participants professed to experiencing involuntary flashbacks during wakefulness (Zeman et al., 2015). So the existence of aphantasics with psychological disorders, trauma-based ones in particular, can be explained on this account. On the face of it, that is a significant benefit that the volitional account has over the metacognitive one. Similar claims have been made about the capacity of volitional account to explain how it is that aphantasics report dreams, since dreaming is an imagery-based process that mostly occurs involuntarily (Whiteley, 2021).

Yet the volitional account is not without difficulties. Indeed, some imagery measures on which aphantasics return poor results appear to measure involuntary, intrusive imagery. For instance, the 12-point questionnaire constituting the Spontaneous Use of Imagery Scale (SUIS) asks participants to rate their agreement with sentences like ‘When I first hear a friend’s voice, a visual image of him or her almost always springs to mind.’ (Reisberg et al., 2003) The use of ‘springs’ here, plus ‘spontaneous’ in the SUIS’s title, indicates that the scale measures involuntary, intrusive imagery. Crucially, insofar as aphantasics have been found to perform poorly on both the SUIS, which appears to measure involuntary imagery, and VVIQ, which appears to measure voluntary imagery (Keogh and Pearson, 2018), this may be thought to lend support to the third account of aphantasia mentioned earlier, i.e., as the complete absence of imagery (Keogh and Pearson, 2018; Wicken et al., 2021).

However, the fact that aphantasics return poor results on the SUIS and VVIQ is not necessarily evidence for understanding aphantasia as a thoroughgoing lack of imagery. The problem is the SUIS. A shortcoming of this measure is that many of its questions are ambiguous between readings on which the imagery measured is involuntary or voluntary. For instance, only one other question, in addition to the one mentioned above about the friend’s voice, strongly implies involuntary imagery: ‘If I catch a glance of a car that is partially hidden behind bushes, I automatically ‘complete it,’ seeing the entire car in my mind’s eye.’ Yet even this question may be read as referring to voluntary imagery. In addition, the idea that one ‘completes’ occluded parts of objects with imagery, rather than other psychological resources, is not a universally agreed principle (Briscoe, 2011). So a denial that one uses imagery to ‘complete’ objects may be offered even by those with normal imagery capacities.

The remaining ten questions on the SUIS might be taken to refer either to voluntary or involuntary imagery. One talks about having a ‘clear picture’ in mind when thinking about visiting a relative. This seems neutral between whether the imagery is voluntary or involuntary. Another mentions ‘finding oneself picturing’ what a radio announcer looks like. But insofar as ‘picturing’ is an active verb, subjects may take this to refer to a deliberate, voluntary act rather than something that occurs involuntarily or spontaneously. All other questions, bar one, ask about situations in which one ‘visualizes.’ Like ‘picturing’, the verb ‘visualizes’ may also be taken to imply a voluntary act rather than something that occurs involuntarily. In sum, a low score on the SUIS does not necessarily evidence that an individual lacks involuntary imagery and so is not, in combination with a low score on the VVIQ, evidence for the thoroughgoing view that aphantasics lack all imagery.

In terms of psychopathology, the thoroughgoing account pictures aphantasia as offering greater protection against psychopathology than does the metacognitive account. For the account says that aphantasia is the complete absence of imagery, both voluntary and voluntary. But in this, the view risks making the existence of mental health conditions among aphantasics as puzzling as on the metacognitive account. Although there may be other issues on which it will be discovered that the metacognitive and thoroughgoing accounts have an explanatory edge over the volitional account, in terms of making sense of psychopathology among aphantasics, here the volitional account appears to have an explanatory advantage.

## Future directions

The relationship between aphantasia and psychopathology is a significant arena for future psychological research insofar as it bears on both the heterogeneity of human cognition and the effective treatment of psychological disorders in neurodiverse populations. Future research into aphantasia in general and its relation to psychopathology in particular may benefit from:

1. *Distinguishing between intra-aphantasic groups, and not just between aphantasics and non-aphantasics, when enquiring into mental health histories*. Aphantasia is not a completely unitary condition, given the distinction between congenital and non-congenital (psychogenic and neurogenic) aphantasia. So in order to arrive at a more detailed picture of the relationship between the condition and susceptibility to psychological disorder, intra-group differences among aphantasics should be examined too. Indeed, the mental health histories of congenital and non-congenital aphantasics should be expected to differ, given that congenital aphantasics have a lifelong imagery deficit while at least some non-congenital aphantasia (psychogenic aphantasia) is believed to be caused by psychological disorder.
2. *Assessing whether psychopathology in aphantasics is treatable via imagery-based interventions*. Assessing conditions such as PTSD, major depressive disorder and bipolar disorder may be particularly illuminating insofar as imagery-based therapies have proven effective treatments for all three in non-aphantasics (Smucker et al., 1995; Brewin et al., 2009; Arntz, 2012; Holmes et al., 2016). If imagery interventions are found not to be effective for aphantasic individuals with these conditions, as is likely, then at least two explanations are possible. First, it is possible that non-image-based styles of cognition are at work in maintaining psychological disorder in aphantasics. That is, despite what has been argued here, it is open that aphantasics experience psychopathology differently than non-aphantasics; that is, *via* exclusively verbal styles of cognition, e.g., *via* verbal rumination and delusion only, with no imagery at all, whether voluntary or involuntary. Another explanation for the potential failure of imagery-based interventions to treat psychopathology in aphantasics is that many of these require the subject’s voluntary manipulation of their imagery (Introduction), e.g., replacing negative imagery with more positive images. Again, VVIQ results of aphantasic individuals suggest that voluntary imagery is precisely the kind of imagery that aphantasics lack.
3. *Assessing aphantasia via a questionnaire which unequivocally concerns involuntary imagery*. As argued above, the SUIS does not substantively differ from the VVIQ insofar as the majority of its questions may be read as referring to voluntary imagery. Being able to assess aphantasia *via* a questionnaire which unequivocally concerns the patient’s capacity for involuntary imagery is crucial for the issue of how best to define aphantasia. After all, the volitional account predicts that aphantasics do not substantially differ from non-aphantasics in terms of capacity for this kind of imagery. Moreover, although as mentioned there exist psychophysical correlates of aphantasia *via* which the condition may be tested for ‘objectively’ (Keogh and Pearson, 2018; Wicken et al., 2021), the value of subjective measures should not be overlooked. These may provide important insights into how the minds of aphantasics work and how those with imagery deficits experience the world. Modifying existing interview schedules and studies related to obsessive–compulsive disorder (Speckens et al., 2007; Lipton et al., 2010) may be useful in this regard insofar as these often focus on involuntary imagery.
4. *Determining whether there are differences between aphantasics and non-aphantasics, and between congenital and non-congenital (psychogenic and neurogenic) aphantasics, in terms of history of addiction*. Like various psychopathologies, addictions are also thought to be partially maintained by dysfunctions of mental imagery, many of which also concern patterns of involuntary, intrusive imagery, i.e., of the object of addiction or future satisfaction (Andrade et al., 2012; May et al., 2015). Thus, research into aphantasia and psychopathology may benefit from examining the susceptibility of aphantasics to addiction in tandem with their susceptibility to psychopathology insofar as parallel results should be expected. Research here will benefit from differentiating distinct addictions, rather than enquiring into history of addiction *per se*, as well as comparing addictions in congenital and non-congenital (psychogenic and neurogenic) aphantasics and not just between aphantasics and non-aphantasic controls. Again, findings here may be relevant for identifying effective treatments insofar as addictions in congenital aphantasics may be driven, at least in part, by uniquely verbal cognitive styles, entailing that imagery-based interventions on addiction in aphantasics may prove less effective than those undertaken by non-aphantasics.
5. *Determining the susceptibility of those with hyperphantasia to maladaptive daydreaming and related conditions*. Individuals with so-called ‘hyperphantasia’ lie at the opposite end of the spectrum from aphantasics, having ‘an abundance’ (Zeman et al., 2020, p. 426) of mental imagery. As with the nature of the imagery deficit constitutive of aphantasia, various questions can be asked about this ‘abundance’. Is hyperphantasia an abundance of vivacity, frequency, maintenance or voluntary control, etc. with respect to imagery? That issue aside, research into hyperphantasia is even more embryonic than research into aphantasia, but one hypothesis that bears investigating is whether the condition is a risk factor for conditions like maladaptive daydreaming. Maladaptive daydreaming is partially characterized by episodes of highly vivid imagery, sometimes initiated voluntarily but sometimes occurring intrusively, that can absorb patients for up to hours at a time (Somer, 2002; Bigelsen et al., 2016). Insofar as maladaptive daydreaming shows signs of being strongly-related to obsessive–compulsive disorder (Salomon-Small et al., 2021) hyperphantasia may pose a dual if not multifaceted risk for psychopathology. Indeed, consistent with the literature canvassed in “Introduction” that suggested imagery is a significant contributor to psychological disorder, higher levels of imagery vivacity are associated with bipolar disorder (Peckham et al., 2020), fantasy proneness (Aleman and de Haan, 2004) and schizophrenia (Van De Sack et al., 2005), among others. So hyperphantasic individuals may be especially at risk of these psychopathologies as well. On the other hand, imagery-based therapies may prove to be an especially effective counter to psychopathology in this population, another hypothesis that bears investigating. Exactly similar hypotheses regarding elevated risk, but also increased responsiveness to imagery-based treatments, for addiction might also be considered.

## Data availability statement

The original contributions presented in the study are included in the article/supplementary material, further inquiries can be directed to the corresponding author.

## Author contributions

The author confirms being the sole contributor of this work and has approved it for publication.

## Funding

Open access fees to be covered by the Open University Fellowship Academy funds.

## Conflict of interest

The author declares that the research was conducted in the absence of any commercial or financial relationships that could be construed as a potential conflict of interest.

## Publisher’s note

All claims expressed in this article are solely those of the authors and do not necessarily represent those of their affiliated organizations, or those of the publisher, the editors and the reviewers. Any product that may be evaluated in this article, or claim that may be made by its manufacturer, is not guaranteed or endorsed by the publisher.

## Footnotes

^1^This possibility was suggested to me by one of the journal’s referees.
